# Association between depression and low skeletal muscle mass in patients with newly diagnosed lung cancer

**DOI:** 10.3389/fpsyg.2026.1829410

**Published:** 2026-05-29

**Authors:** Zeynep Alaca Topcu, Ismail Yurtsever, Zehra Sucuoglu Isleyen, Mehmet Besiroglu, Atakan Topcu, Ayse Irem Yasin, Abdallah Tm Shbair, Melih Simsek, Mesut Seker, Mahmut Gumus, Haci Mehmet Turk

**Affiliations:** 1 Department of Medical Oncology, Istanbul Medeniyet University Göztepe Prof. Dr. Süleyman Yalçın City Hospital, Istanbul, Türkiye; 2 Department of Radiology, Bezmialem Vakif University Hospital, Istanbul, Türkiye; 3 Department of Medical Oncology, Bezmialem Vakif University Hospital, Istanbul, Türkiye

**Keywords:** Beck Depression Inventory, Charlson Comorbidity Index, depression, lung cancer, sarcopenia, skeletal mass index, skeletal muscle mass

## Abstract

**Background:**

Depression and reduced muscle mass are common in patients with lung cancer, and both are associated with adverse clinical outcomes. However, data examining their interrelationship remain limited.

**Materials and methods:**

This cross-sectional study included 100 patients with newly diagnosed lung cancer undergoing systemic therapy. Depressive symptoms were assessed using the Beck Depression Inventory (BDI). Skeletal muscle mass was evaluated using computed tomography (CT)-derived skeletal muscle index (SMI) at the L3 vertebral level. Clinical and demographic variables were analyzed for associations with depression.

**Results:**

Clinically relevant depressive symptoms were identified in 71% of patients, and CT-defined low skeletal muscle mass (LSMM) was present in 67% of patients. Scores on BDI were significantly and inversely correlated with total muscle area (TMA; *r* = −0.418, *p* < 0.001) and skeletal muscle index (*r* = −0.358, *p* < 0.001). In age-adjusted multivariate analysis, low SMI (OR 3.05, 95% CI 1.15–8.09) and higher Charlson Comorbidity Index (CCI) values (OR 3.27, 95% CI 1.25–8.68) were independently associated with depression.

**Conclusion:**

Depressive symptoms are highly prevalent in lung cancer and are independently associated with CT-defined LSMM and a higher comorbidity burden. Routine assessment of SMI on staging CT scans may help identify patients at increased risk and support early multidisciplinary interventions.

## Introduction

Lung cancer remains one of the leading causes of cancer-related morbidity and mortality worldwide, with a substantial proportion of patients presenting at an advanced stage and requiring systemic therapy (Bray et al., 2024). Beyond the tumor burden and treatment-related toxicity, patients with advanced lung cancer frequently experience a range of physical and psychological comorbidities that significantly affect treatment tolerance, quality of life, and survival outcomes (Charlson et al., 1987; Fearon et al., 2011; Shachar et al., 2016; Pinquart and Duberstein, 2010).

Depression is highly prevalent among patients with lung cancer, with reported prevalence rates reaching up to one-third of patients and exceeding 50% in certain cohorts, particularly among younger individuals and those with advanced disease (Hopwood and Stephens, 2000; Yan et al., 2019). Notably, depression is closely associated with a greater symptom burden, functional impairment, and poorer clinical outcomes. Despite increasing recognition of its clinical significance, recent bibliometric analyses highlight persistent gaps in understanding the complex clinical and biological interplay between depression and lung cancer (Lin et al., 2025). The presence of depressive symptoms has been associated with poorer adherence to systemic therapy, decreased functional capacity, impaired quality of life, and worse survival outcomes (Pinquart and Duberstein, 2010; Satin et al., 2009; DiMatteo et al., 2000). Notably, depression in patients with cancer is often underrecognized, as its symptoms frequently overlap with cancer-related fatigue, anorexia, and weight loss (Massie, 2004).

Sarcopenia is highly prevalent in patients with lung cancer and has been shown to be an independent predictor of poorer overall survival across different histological subtypes and disease stages (Yang et al., 2019). Low skeletal muscle mass (LSMM) is highly prevalent among patients with lung cancer, affecting approximately 40–45% of patients—particularly in Asian populations—and has been consistently associated with poorer survival outcomes, including reduced overall and recurrence-free survival (Tao et al., 2026). In patients with advanced lung cancer, sarcopenia is highly prevalent and has been associated with increased chemotherapy-related toxicity, more frequent dose reductions, treatment interruptions, and reduced survival (Shachar et al., 2016; Baracos et al., 2010; Prado et al., 2007). Computed tomography (CT)-based assessment of skeletal muscle mass at the lumbar vertebral level has become a widely accepted method for identifying sarcopenia in clinical oncology research (Mourtzakis et al., 2008).

Growing evidence indicates a bidirectional relationship between sarcopenia and depression (Soysal et al., 2017; Zhang et al., 2025). Biologically, chronic systemic inflammation, dysregulation of the hypothalamic–pituitary–adrenal axis, and elevated pro-inflammatory cytokines, including interleukin 6 and tumor necrosis factor alpha, have been implicated in the pathogenesis of both conditions (Miller and Raison, 2016; Silverman and Sternberg, 2012). In addition, sarcopenia-related functional decline, fatigue, and loss of independence may increase the risk of depressive symptoms, while depression can further accelerate muscle loss through reduced physical activity, poor nutrition, and neuroendocrine changes (Willbanks et al., 2024).

In recent years, increasing attention has been directed toward the concept of sarcopenic obesity, defined as the coexistence of reduced skeletal muscle mass and increased adiposity (Jurdana and Cemazar, 2024). This condition is increasingly recognized in patients with cancer and has important clinical implications, as it may remain underdiagnosed when body mass index (BMI) alone is considered. Growing evidence suggests that sarcopenic obesity is associated with adverse outcomes, including reduced treatment tolerance, increased toxicity, impaired physical function, and shorter survival. Importantly, it reflects a complex interplay between metabolic dysregulation, systemic inflammation, and cancer-related cachexia (Jiang et al., 2025). Despite its emerging significance, sarcopenic obesity remains insufficiently characterized in patients with lung cancer, highlighting the need for further investigation into its clinical and prognostic relevance (Wang et al., 2025).

However, despite the established clinical relevance of both sarcopenia and depressive symptoms in lung cancer, data specifically examining their association using objective CT-based muscle measurements in patients with newly diagnosed advanced disease remain limited. Although the individual prognostic significance of depression and sarcopenia has been increasingly recognized, their interrelationship—particularly among patients scheduled to receive systemic therapy—has not been adequately investigated. Understanding the potential association between sarcopenia and depression may provide valuable insights for comprehensive patient assessment and facilitate the identification of high-risk subgroups who could benefit from early multidisciplinary interventions, including nutritional, physical, and psychological support. Given the growing global burden of lung cancer, identifying patients at an increased risk for depression is of substantial clinical importance. Therefore, the present study aims to explore the association between CT-defined LSMM and depressive symptoms in patients newly diagnosed with advanced lung cancer prior to the initiation of systemic therapy.

## Materials and methods

Between October 2022 and February 2023, a cross-sectional study was conducted in the medical oncology setting of tertiary care academic institutions. Due to the cross-sectional design of the study, causal relationships between sarcopenia and depressive symptoms cannot be inferred. Patients with stage 2–3 (locoregional) and stage 4 (metastatic) lung cancer undergoing systemic treatment were eligible for inclusion. The exclusion criteria were as follows: Eastern Cooperative Oncology Group (ECOG) Performance Status (PS) > 2, the presence of concomitant malignancies, significant visual or hearing impairments that prevented completion of the questionnaire despite family assistance, severe psychiatric disorders, an estimated life expectancy of less than 3 months, or enrollment in hospice care. Patients who met the eligibility criteria were enrolled in the study. The study was approved by the Clinical Research Ethics Committee of Bezmialem Vakif University (Approval No: 2020/330). The studies were conducted in accordance with local legislation and institutional requirements. Written informed consent was obtained from all participants prior to participation in the study.

### Sociodemographic and clinical factors

Baseline demographic and clinical characteristics were collected through face-to-face interviews at study enrollment. Body mass index (BMI) was calculated as weight (kg) divided by height squared (m^2^). Performance status was assessed using the five-point activity scale of the ECOG.

### Charlson Comorbidity Index (CCI)

The comorbidity burden was evaluated using the Charlson Comorbidity Index (CCI), a validated prognostic tool that estimates 1-year mortality risk based on the presence and weighted scoring of predefined comorbid conditions (Charlson et al., 1987; Quan et al., 2011). Each comorbidity is assigned a weight ranging from 1 to 6 according to its associated risk of mortality, and a total CCI score is calculated for each patient. Higher CCI scores indicate a greater comorbidity burden and an increased risk of mortality. The CCI was incorporated into the analyses to adjust for baseline comorbidity status. Receiver operating characteristic (ROC) curve analysis was performed to determine the optimal cutoff value of the CCI in the study population.

Depressive symptoms were assessed during the treatment decision-making or initiation phase, prior to the administration of systemic therapy. Therefore, patients had not yet received chemotherapy at the time of evaluation, minimizing the potential confounding effect of treatment on depressive symptoms.

### Assessment of depressive disorder

Depressive symptoms were measured using the Beck Depression Inventory (BDI), a widely used 21-item self-report questionnaire designed to determine the severity of depressive symptoms (Beck, 1961). Each item is rated on a 4-point scale from 0 to 3, yielding a total score ranging from 0 to 63, with higher scores indicating greater severity of depression. The BDI was administered through face-to-face interviews during the study period while patients were undergoing systemic treatment. Depression severity was categorized according to total BDI scores as follows: 0–9, no depression; 10–16, mild depression; 17–29, moderate depression; and 30–63, severe depression (Beck et al., 1996). Patients with a BDI score of ≥10 were classified as having depression. In this study, “depressive symptoms: refer to clinically relevant symptoms assessed using the BDI rather than a formal psychiatric diagnosis.

### Sarcopenia assessment

Computed tomography (CT) images obtained prior to the initiation of systemic therapy were used to assess CT-defined low skeletal muscle mass. For this purpose, abdominal CT performed as part of routine staging—complementing thoracic CT—was utilized when available. All analyses were based on routinely acquired staging imaging, and no additional radiological examinations were performed for the purposes of this study. All scans were independently analyzed by an experienced radiologist (IY) who was blinded to patients’ clinical, laboratory, and psychological data. All measurements were performed by the same radiologist to minimize interobserver variability. A single cross-sectional image at the level of the third lumbar vertebra (L3) was used as the standard anatomical landmark, given its strong correlation with whole-body skeletal muscle mass. Skeletal muscle area at L3—including the psoas, paraspinal, and abdominal wall muscles—was reported as total muscle area (TMA, cm^2^). Skeletal muscle index (SMI, cm^2^/m^2^) was calculated by normalizing TMA to height squared (SMI = TMA/height^2^) (Mourtzakis et al., 2008). Low skeletal muscle mass was defined using previously established CT-based cutoffs, sarcopenia was defined as SMI ≤ 39 cm^2^/m^2^ for women and SMI ≤ 55 cm^2^/m^2^ for men (Tao et al., 2026). In accordance with the EWGSOP2 consensus, we acknowledge that the assessment of muscle strength and physical performance was not available; therefore, sarcopenia could not be formally diagnosed, and the present study specifically evaluates CT-defined LSMM (Yang et al., 2019).

### Data analysis

Statistical analyses were conducted using SPSS version 24.0 (SPSS Inc., Chicago, IL, USA). Categorical variables were presented as frequencies and percentages, whereas continuous and ordinal variables were reported as mean ± standard deviation or median (range), as appropriate. Data distribution was assessed using the Kolmogorov–Smirnov test. Categorical variables were compared using the Pearson Chi-squared (*χ*^2^) test, and continuous variables were compared using analysis of variance (ANOVA), when applicable. Patient characteristics were summarized using descriptive statistics. Correlations between muscle-related parameters and depression scores were analyzed using Spearman’s rank correlation. Variables with a *p*-value of <0.1 in univariate analyses were entered into a multivariate logistic regression model, which was performed using the backward elimination method to identify independent risk factors for depression. For regression analyses, SMI was dichotomized using the median value of the study population. An ROC analysis was performed to determine the sensitivity and specificity of the Charlson Comorbidity Index (CCI) in predicting depression. Statistical significance was set at a *p*-value of < 0.05.

## Results

A total of 100 patients were enrolled. The median age was 62 years (range, 25–80), with 44% of participants aged ≥65 years. A total of 18 patients (18%) were female, while 82 (82%) of them were male. The mean body mass index was 26.25 ± 4.13 kg/m^2^. A majority of patients were married (81%), and 67% had at least one comorbidity. An ECOG PS of 0–1 was observed in 74% of patients, whereas 26% of patients had a score of 2. At diagnosis, 66% of patients had metastatic disease and 34% had locoregional disease. Histologically, 74% had non-small cell lung cancer and 26% had small cell lung cancer. Clinically relevant depressive symptoms (hereafter referred to as depression) were identified in 71 patients (71%) in the study cohort. CT-defined LSMM was present in 67% of patients. Regarding the comorbidity burden, the mean Charlson Comorbidity Index (CCI) score was 7.41 ± 2.43. The mean total muscle area was 134.99 ± 30.46, and the mean skeletal muscle index was 47.41 ± 9.76. The mean Beck Depression Inventory score was 15.75 ± 10.08. Female sex was significantly more prevalent in the depressed group than in the non-depressed group (18% vs. 0%, *p* = 0.003). Patients with depression were older, with a higher median age (64 vs. 60 years, *p* = 0.013) and a greater proportion of patients aged ≥65 years (36% vs. 8%, *p* = 0.035).

In this study, CT-defined LSMM was significantly more prevalent in the depressed group than in the non-depressed group (52% vs. 15%, *p* = 0.038). Depressed patients also had a higher Charlson Comorbidity Index value (7.83 vs. 6.38, *p* = 0.006). In terms of body composition, depressed patients exhibited lower total muscle area (127.59 vs. 153.10, *p* < 0.001) and skeletal muscle index (45.68 vs. 51.66, *p* = 0.005). As expected, mean Beck Depression Inventory scores were markedly higher in the depressed group (20.0 vs. 5.34, *p* < 0.001). Moreover, no significant differences were observed between depressed and non-depressed patients for the other parameters listed in Table 1, which summarizes the demographic and clinical characteristics of both groups.

**Table 1:** Baseline demographic and clinical characteristics of the study population by depression status.

Variable	Total (*n* = 100)	Non-depressed (*n* = 29)	Depressed (*n* = 71)	*p*-value
Sex, *n* (%)				0.003
Female	18 (18)	0 (0)	18 (18)	
Male	82 (82)	29 (29)	53 (53)	
Age, years, median (range)	62 (25–80)	60 (25–77)	64 (37–80)	**0.013**
Age, years, *n* (%)				0.035
≥65	44 (44)	8 (8)	36 (36)	
<65	56 (56)	21 (21)	35 (35)	
BMI, mean ± SD	26.25 ± 4.13	27.03 ± 3.31	25.93 ± 4.41	0.229
Marital status, *n* (%)				0.396
Married	81 (81)	25 (25)	56 (56)	
Not married	19 (19)	4 (4)	15 (15)	
Comorbidity, *n* (%)				0.503
Yes	67 (67)	18 (18)	49 (49)	
No	33 (33)	11 (11)	22 (22)	
ECOG PS, *n* (%)				0.075
0–1	74 (74)	25 (25)	49 (49)	
2	26 (26)	4 (4)	26 (26)	
Family history of lung cancer, *n* (%)				0.585
Yes	17 (17)	4 (4)	13 (13)	
No	83 (83)	25 (25)	58 (58)	
Smoking, *n* (%)				0.080
Yes	93 (93)	29 (29)	64 (64)	
No	7 (7)	0 (0)	7 (7)	
Alcohol use, *n* (%)				0.953
Yes	28 (28)	8 (8)	20 (20)	
No	72 (72)	21 (21)	51 (51)	
Stage category, *n* (%)				0.054
Locoregional	34 (34)	14 (14)	20 (20)	
Metastatic	66 (66)	15 (15)	51 (51)	
Histology, *n* (%)				0.202
NSCLC	74 (74)	24 (24)	50 (50)	
SCLC	26 (26)	5 (5)	21 (21)	
CT-defined LSMM, *n* (%)				0.038
Yes	67 (67)	15 (15)	52 (52)	
No	33 (33)	14 (14)	19 (19)	
Charlson Comorbidity Index, mean ± SD	7.41 ± 2.43	6.38 ± 2.48	7.83 ± 2.3	**0.006**
Number of prescription drugs, median (range)	2 (0–12)	2 (0–12)	2 (0–10)	0.935
TMA, mean ± SD	134.99 ± 30.46	153.10 ± 31.02	127.59 ± 27.13	**<0.001**
SMI, mean ± SD	47.41 ± 9.76	51.66 ± 10.77	45.68 ± 8.81	**0.005**
BDI, mean ± SD	15.75 ± 10.08	5.34 ± 3.21	20.0 ± 8.74	**<0.001**

BMI, Body mass index; CCI, Charlson Comorbidity Index; ECOG PS, Eastern Cooperative Oncology Group Performance Status; SD, Standard deviation; CT, Computed tomography; LSMM, Low skeletal muscle mass; SCLC, Small cell lung cancer; NSCLC, Non-small cell lung cancer; BDI, Beck Depression Index; TMA, Total cross-sectional skeletal muscle area; SMI, Skeletal muscle index.

Bold values indicate statistically significant results (*p* < 0.05).

A statistically significant negative correlation was observed between Beck Depression Inventory scores and total cross-sectional skeletal muscle area (TMA) (*r* = −0.418, *p* < 0.001). Similarly, Beck Depression Inventory scores were inversely correlated with skeletal muscle index (SMI) (*r* = −0.358, *p* < 0.001), indicating that greater depression severity was associated with lower muscle mass. Table 2 shows the correlation between the BDI, TMA, and SMI.

**Table 2:** Correlation between Beck Depression Inventory scores and total cross-sectional skeletal muscle area and skeletal muscle index.

Variable	TMA (cm^2^)		SMI (cm^2^/m^2^)	
	*r*	*p*-value	*r*	*p*-value
BDI	−0.418	**<0.001**	−0.358	**<0.001**

TMA, Total cross-sectional skeletal muscle area; SMI, Skeletal muscle index; BDI, Beck Depression Inventory.

Bold values indicate statistically significant results (*p* < 0.05).

In the univariate logistic regression analysis, low TMA, low SMI, older age, CCI, ECOG PS, and disease stage were identified as potential risk factors for depression. In the multivariate analysis, low SMI (≤47.7 cm^2^/m^2^) remained independently associated with depression (OR = 3.30, 95% CI: 1.28–8.48; *p* = 0.013). In addition, a higher CCI value (≥6.5) was independently associated with depression (OR = 2.81, 95% CI: 1.11–7.13; *p* = 0.029). After adjusting for age, both low SMI (adjusted OR = 3.05, 95% CI: 1.15–8.09; *p* = 0.025) and a higher CCI value (≥6.5) (adjusted OR = 3.27, 95% CI: 1.25–8.68; *p* = 0.016) remained significantly associated with depression, whereas the other variables were not statistically significant. The results of the univariate and multivariate logistic regression analyses evaluating factors associated with depression are presented in Table 3.

**Table 3:** Univariate logistic regression analysis and multivariate logistic regression analysis of risk factors for depression (Beck Depression Inventory).

Variable	Univariate OR (95% CI)	*p*-value	Multivariate OR (95% CI)	*p*-value	Multivariate Age-adjusted OR (95% CI)	*p*-value
TMA (cm^2^)						
≤133	3.2 (1.28–8.06)	0.013				
>133						
SMI (cm^2^/m^2^)						
≤47.7	3.03 (1.21–7.59)	0.018	3.30 (1.28–8.48)	**0.013**	3.05 (1.15–8.09)	**0.025**
>47.7						
Age						
≥65	3.2 (1.28–8.06)	0.013				
<65						
CCI						
<6.5	2.73 (1.18–6.67)	0.027	2.81 (1.11–7.13)	**0.029**	3.27 (1.25–8.68)	**0.016**
≥6.5						
ECOG PS						
0–1	2.8 (0.87–9.03)	0.084				
2						
Stage						
Locoregional	2.38 (0.97–5.81)	0.057				
Metastatic						

TMA, Total cross-sectional skeletal muscle area; SMI, Skeletal muscle index; CCI, Charlson Comorbidity Index; ECOG PS, Eastern Cooperative Oncology Group Performance Status; OR, Odds ratio; SD, Standard deviation; CI, Confidence interval.

Bold values indicate statistically significant results (*p* < 0.05).

## Discussion

In this cross-sectional study, depression was highly prevalent among patients with newly diagnosed lung cancer, affecting 71% of the cohort. The relatively high prevalence of depression observed in our cohort may be attributable to the use of a symptom-based screening tool rather than a diagnostic interview, as well as to the partial overlap between somatic depressive symptoms and cancer-related symptoms. Although depression was numerically more frequent among patients with metastatic disease, the difference did not reach statistical significance. In addition, CT-defined LSMM was identified in 67% of patients. A significant correlation was observed between depression scores and the sarcopenia-related parameters, namely TMA and SMI. Lower TMA and SMI values were associated with higher depression scores. Furthermore, potential risk factors for depression were evaluated, and low SMI and a high CCI value, reflecting a greater comorbidity burden, were identified as factors independently associated with depression. Our findings demonstrate that both depression and CT-defined LSMM are highly prevalent among patients with lung cancer undergoing chemotherapy. Moreover, depression was significantly correlated with sarcopenia-related parameters. These results suggest that depression should not be overlooked, particularly in patients with low SMI and a high comorbidity burden. Early recognition and appropriate management of depression in this vulnerable population may be crucial for improving treatment adherence, quality of life, clinical outcomes, and overall patient care.

Depression is known to occur at a substantial rate in patients with lung cancer (Hopwood and Stephens, 2000; Lin et al., 2025). Although somatic symptoms may overlap with cancer-related complaints, the BDI has been widely used in oncology populations (Beck et al., 1996). Moreover, previous large-scale studies have suggested that depression may be independently associated with poor prognosis in patients with cancer, underscoring the clinical importance of early recognition and the identification of factors contributing to depression (Satin et al., 2009; Walker et al., 2021; Walker et al., 2020). Sarcopenia is also common in many cancers, including lung cancer, and may be associated with impaired function, reduced quality of life, and adverse clinical outcomes. Biologically, skeletal muscle functions as a metabolic system that produces myokines capable of modulating neuroinflammation and neuroplasticity (Pedersen and Febbraio, 2012; Severinsen and Pedersen, 2020). Muscle loss may, therefore, enhance pro-inflammatory signaling while attenuating protective neuromodulatory pathways—mechanisms implicated in both cancer cachexia and depression (Miller and Raison, 2016; Eyre and Baune, 2012). From a functional perspective, sarcopenia results in reduced physical activity, impaired independence, and social withdrawal, all of which are well-recognized contributors to depression symptomatology (Schuch et al., 2018).

Patients with depression often participate less in physical activity, representing a key pathway leading to sarcopenia (Budui et al., 2015; Hallgren et al., 2016; Booth et al., 2012). Clinical stressors such as chronic comorbidities, hospitalization, and poor nutritional status can both trigger and exacerbate depression (Cole and Dendukuri, 2003; Alexopoulos, 2005). Collectively, these factors may constitute shared mechanisms underlying the association between depression and muscle-wasting syndromes, including sarcopenia and cachexia. The coexistence of sarcopenia and depression has been increasingly reported in oncology. Prior studies have shown that sarcopenic cancer patients experience higher rates of depression, fatigue, and poorer quality of life and that physical frailty and muscle wasting are associated with emotional distress in lung cancer (Nipp et al., 2018; Lehto, 2017). A recent systematic review and meta-analysis further confirmed a significant association between sarcopenia and depression across diverse populations (Li et al., 2022; Chang et al., 2017). Our findings, consistent with recent studies highlighting the importance of this field, underscore the clinical relevance of evaluating body composition in lung cancer patients, given the frequent co-occurrence of sarcopenia and depression (Alexopoulos, 2005). In our cohort, depression was associated with lower TMA and SMI values, with BDI scores inversely correlated with both. Low SMI and a high CCI value were independently associated with depression, suggesting a possible biological association between sarcopenia, comorbidity, and depression. Although older age was associated with depression in the univariate analysis, this association lost significance after adjusting for SMI, indicating that muscle loss may better reflect biologically relevant aging than chronological age. A higher comorbidity burden also independently predicted depression, defining a particularly vulnerable subgroup when accompanied by low muscle mass (Xue et al., 2022). A meta-analysis examining the association between multimorbidity and depression reported a twofold to threefold increased risk of depression among individuals with multiple chronic conditions compared to those without multimorbidity (Read et al., 2017). Similarly, a population-based study demonstrated a positive association between higher CCI scores and depression risk, along with increased all-cause mortality among individuals with depression (Wang et al., 2024). Furthermore, a recent population-based study suggested that the CCI may mediate the relationship between body mass index and depression, indicating that the improved management of chronic conditions may reduce depression risk (Liu et al., 2025). Consistent with these findings, our study demonstrated a significant association between a higher comorbidity burden and depression among patients with lung cancer undergoing chemotherapy; those with a CCI of ≥6.5 had a 3.27-fold higher likelihood of depression. Evidence regarding the relationship between the CCI and depression in oncology populations remains limited. Therefore, our results provide additional insights into the role of the comorbidity burden in the development of depressive symptoms and support the integration of systematic comorbidity assessment into the psychosocial evaluation of this vulnerable population.

Although we used widely accepted international SMI cutoffs to ensure comparability with prior studies, emerging evidence suggests that disease- and stage-specific thresholds may provide superior prognostic discrimination. A large Turkish cohort of patients with stage III NSCLC proposed lower, disease-specific cutoffs and demonstrated stronger associations with clinical outcomes (Gumustepe et al., 2025). However, such thresholds are typically derived from more homogeneous populations, and their generalizability across heterogeneous cohorts remains uncertain. In addition, ethnic and population-specific variability may further influence skeletal muscle mass. Therefore, tailored cutoffs may improve prognostic accuracy in selected populations.

Notably, ECOG performance status and disease stage were not independently associated with depression, suggesting that body composition parameters may identify dimensions of vulnerability not reflected by conventional clinical indices. Clinically, a routine assessment of SMI using standard staging CT scans may help identify lung cancer patients at an increased risk of depression and support early referral for multidisciplinary interventions, including nutritional, physical, and psycho-oncological support. This study has several limitations. First, its cross-sectional design precludes conclusions about causality. Second, the BDI assesses symptom severity rather than formal psychiatric diagnoses and may overlap with cancer-related symptoms. Third, potential confounders, including inflammatory markers and nutritional status, were not comprehensively evaluated. Finally, sarcopenia was assessed solely based on muscle quantity using CT-derived L3 skeletal muscle index (SMI), while muscle strength and physical performance—both key components of contemporary sarcopenia definitions—were not evaluated. Therefore, our findings reflect low skeletal muscle mass, which represents an important but not comprehensive component of sarcopenia, rather than fully defined sarcopenia.

## Conclusion

In conclusion, depression is highly prevalent in patients with lung cancer and is independently associated with low SMI and high CCI values, reflecting an increased comorbidity burden. CT-defined LSMM, assessed on routine staging CT scans, may serve as a readily available imaging biomarker of psychological vulnerability, highlighting the importance of early risk stratification and multidisciplinary intervention, particularly in patients with substantial comorbidity. Longitudinal studies are needed to further clarify the temporal and causal relationships among these interrelated clinical conditions.

## Data Availability

The raw data supporting the conclusions of this article will be made available by the authors, without undue reservation.
